# Ulcerative Squamous Eyelid Papilloma: A Rare Presentation

**DOI:** 10.18502/jovr.v14i4.5463

**Published:** 2019-10-24

**Authors:** Sharma Reena, Krishna Mani, Sharma Brahma Deo, Khan Asma

**Affiliations:** ^1^Department of Ophthalmology, UP University of Medical Sciences, Saifai, Etawah, Uttar Pradesh, India; ^2^Department of Pathology, UP University of Medical Sciences, Saifai, Etawah, Uttar Pradesh, India

**Keywords:** Carcinoma, Eyelid, Histopathological, Squamous Papilloma

## Abstract

**Purpose:**

To highlight the importance of histopathological evaluation of a lid mass to prognosticate the disease. We report a case of ulcerative squamous cell papilloma with clinical features suggesting malignancy.

**Case report:**

A 65-year-old man presented with a rapidly enlarging mass in the left upper eyelid, with clinical features suggesting a squamous cell carcinoma. However, a repeat histopathological examination showed no malignant cells. The patient was diagnosed with squamous cell papilloma. He was followed-up for 30 months and no recurrence was observed. No such case has previously been reported in the literature.

**Conclusion:**

This report highlights the need for histopathological examination of all eyelid lesions to enable surgeons to prognosticate the disease.

##  INTRODUCTION

Eyelid papilloma is one of the most common eyelid tumors and typically occurs in middle-aged or elderly patients.^[1,2]^ These tumors are papillomatous and appear as a smooth, rounded, or pedunculated elevation of abnormal tissue.

Squamous papilloma may mimic other benign eyelid lesions with the same appearance, and malignant skin lesions, particularly squamous cell carcinoma. Feeder vessels, hyperkeratosis, leukoplakic plaque, keratinization, scaling, and ulceration are considered as features suggestive of malignancy. Nevertheless, atypical malignant behavior of a benign eyelid lesion is unusual. Herein, we report the case of a lesion presenting with typical malignancy features that was histopathologically found to be benign. To the best of our knowledge and based on a review of the literature, ulcerative squamous papilloma of the eyelid has not been reported thus far.

**Figure 1:**
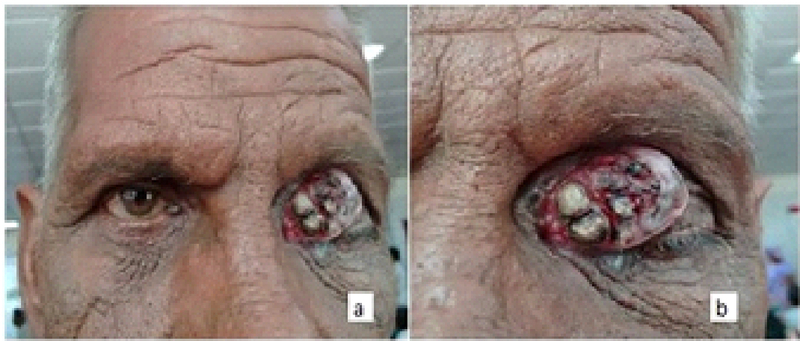
(a) Suspected squamous cell carcinoma in the left upper eyelid. (b) Enlarged view of the same mass.

**Figure 2:**
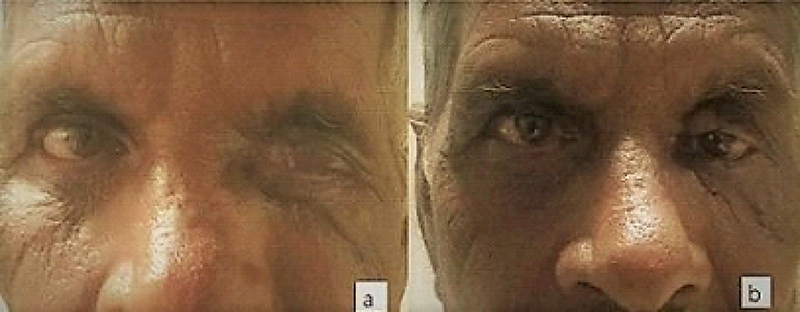
(a) Patient after mass excision and first stage Cutler–Beard surgery. (b) Patient after second stage Cutler–Beard surgery (two weeks).

**Figure 3:**
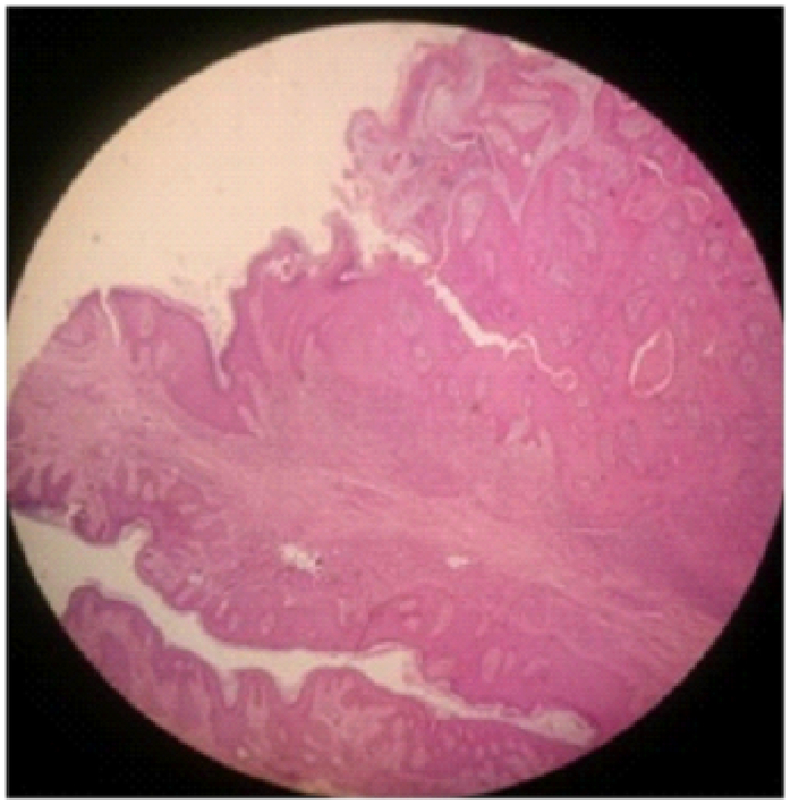
Histopathological section showing papillomatous features and total absence of atypia.

##  CASE REPORT 

A 65-year-old man presented with a mass on the left upper eyelid that had been progressively enlarging for the past seven years. A pedunculated growth measuring 20 × 12 mm was observed involving the medial half of the eyelid. The mass was huge, with superficial ulceration and a red base with sharply defined indurated borders. It was pedunculated, such that the base of the mass was smaller than its superficial appearance. He was diagnosed with papilloulcerative squamous cell carcinoma [Figure 1]. The mass was excised with a 4-mm free margin, creating a defect measuring 8 × 6 cm, which was reconstructed using a Cutler–Beard flap. The flap was prepared from the inferior eyelid below the tarsus to ensure compatibility with the superior eyelid defect by making a horizontal incision 4 mm below the lid margin. The bridge flap was advanced to the superior eyelid below the eyelash margin. The anterior and posterior lamellae were sutured separately to the levator muscle and the rest of the orbicularis muscle by placing the flap on the defect area [Figure 2(a)]. Second stage surgery was performed four weeks postoperatively to separate the superior and inferior eyelids and reconstruct the upper lid margin [Figure 2(b)].

Histopathological examination revealed no atypia; rather, the lesion was composed of papillae with vascularized connective tissue covered by acanthotic epithelium. Hence, it was diagnosed as ulcerative squamous cell papilloma [Figure 3]. A repeat examination with repeat tissue cuts and histopathology slide preparation performed by another pathologist did not change the diagnosis. The patient was followed-up for 30 months and no recurrence was observed.

##  DISCUSSION 

Ocular papillomas favor sites where the epithelium undergoes transition. They can occur at either the limbus or the eyelid margin. Papillomas typically arise from the basal cells but, rarely, may form through the proliferation of squamous cells. Squamous cell papilloma is the most common benign lesion of the eyelid, constituting 13–19.5% of benign eyelid tumors.^[3,4]^

For squamous cell papilloma, having surface ulceration and indurated margins is not unusual. In the present case, the clinical appearance suggested malignancy, but histopathology changed the diagnosis. Kersten et al reported that the majority of the initially benign lesions were found to be malignant on repeat histopathology.^[5]^ Our case revealed an absence of atypia on repeat examination. Furthermore, the patient was followed-up for two and a half years with no recurrence.

Although rare, case reports of surface pigmentation and hyperkeratosis in squamous papilloma exist.^[6,7]^ This also emphasizes the need for histopathological examination of all eyelid lesions to enable surgeons to prognosticate the disease and advise patients accordingly.

The gold standard treatment for eyelid carcinomas is surgical resection with clear margins followed by reconstructive procedures. Cutler–Beard flap surgery is a successful procedure for superior eyelid tumors with wide tissue loss.^[8,9]^ The long-time closure of the eyelids, and the need for a second surgery are the major disadvantages of this procedure.

The patient gave consent for reproduction of the photographs in the journal.

##  Declaration of Patient Consent

The authors certify that they have obtained all appropriate patient consent forms. In the form the patient has given his consent for his images and other clinical information to be reported in the journal. The patient understand that his name and initial will not be published and due efforts will be made to conceal his identity, but anonymity cannot be guaranteed.

##  Financial Support and Sponsorship

Nil.

##  Conflicts of Interest

There is no conflict of interest.
